# Therapeutic Potential of miR-4711-5p in Pancreatic Cancer: Antitumor Activity and Mechanistic Insights

**DOI:** 10.3390/cancers18071104

**Published:** 2026-03-29

**Authors:** Yuhki Yokoyama, Yoshihiro Morimoto, Hiroyuki Yamamoto, Shihori Kouda, Shiho Kawanami, Ruijia Yang, Yingjue Zhang, Manami Tsujimoto, Nanami Nagata, Yuki Shimomura, Kana Nishida, Tsuyoshi Hata, Akira Inoue, Satoshi Shibata, Hirofumi Yamamoto, Masaki Mori

**Affiliations:** 1 Department of Molecular Pathology, Division of Health Sciences, Graduate School of Medicine, The University of Osaka, 1-7, Yamadaoka, Suita 565-0871, Osaka, Japan; yyokoyama@sahs.med.osaka-u.ac.jp (Y.Y.); u437535i@ecs.osaka-u.ac.jp (R.Y.); u626577e@ecs.osaka-u.ac.jp (Y.Z.);; 2 Department of Gastroenterological Surgery, Osaka General Medical Center, 3-1-56 Bandaihigashi, Osaka 558-8558, Osaka, Japan; 3 Department of Surgery, Gastroenterological Surgery, Graduate School of Medicine, The University of Osaka, 2-2, Yamadaoka, Suita 565-0871, Osaka, Japan; 4 Nanobeyond Inc., 7-7-15 Saito-Asagi, Ibaraki 567-0085, Osaka, Japan; 5 Graduate School of Medicine, Tokai University, 143 Shimokasuya, Isehara 259-1193, Kanagawa, Japan; 6 Department of Gastroenterological Surgery, Ikeda City Hospital, 3-1-18 Jyonan, Ikeda 563-8510, Osaka, Japan

**Keywords:** pancreatic cancer, miR-4711-5p, cancer stem cells, safety evaluation, primate

## Abstract

**Background/Aim:** Pancreatic cancer remains one of the most lethal malignancies, with limited therapeutic options and an extremely poor prognosis. MicroRNAs (miRNAs), which regulate gene expression at the post-transcriptional level, have emerged as promising candidates for next-generation cancer therapeutics. The purpose of this study is to clarify the feasibility of miR-4711 as a potential therapeutic option against pancreatic cancer. **Materials and Methods:** The effects of miR-4711-5p were examined in pancreatic cancer cell lines with respect to cell proliferation, apoptosis, cancer stemness, cell cycle progression, and invasive capacity. RNA sequencing and in silico analyses were performed to identify potential target genes of miR-4711-5p. For in vivo safety evaluation, miR-4711-5p was formulated with super carbonate apatite, a delivery vehicle that is already amenable to large-scale production, and administered to cynomolgus monkeys. A nucleic acid dose equivalent to 10 times the effective dose observed in prior mouse efficacy studies was used. General clinical conditions, body weight, food consumption, ophthalmologic findings, electrocardiography, blood pressure, hematological and biochemical parameters, and histopathological changes were systematically assessed. **Results:** miR-4711-5p significantly suppressed cancer stemness, cell proliferation, and invasion, while inducing apoptosis and delaying cell cycle progression in pancreatic cancer cells. RNA sequencing and bioinformatic analyses identified *MET*, *CTSA*, and *ANO1* as potential target genes of miR-4711-5p. In the cynomolgus monkey study, administration of miR-4711-5p formulated with super carbonate apatite resulted in no apparent differences compared with the control group in body weight, clinical observations, laboratory parameters, or histopathological findings, indicating the absence of treatment-related adverse effects even at a supra-therapeutic dose. **Conclusions:** These findings demonstrate that miR-4711-5p exerts potent antitumor effects against pancreatic cancer cells while exhibiting a favorable safety profile in a non-human primate model. Collectively, this study provides strong preclinical evidence supporting miR-4711-5p as a novel and safe therapeutic strategy for pancreatic cancer and represents an important step toward clinical application.

## Simple Summary

Pancreatic cancer remains one of the most lethal malignancies, with limited therapeutic options and an extremely poor prognosis. MicroRNAs have emerged as promising candidates for next-generation cancer therapeutics. Here, we investigated the antitumor effects of miR-4711-5p in pancreatic cancer cells and evaluated its preclinical safety using a scalable nucleic acid delivery system in a non-human primate model. We showed that miR-4711-5p suppressed cancer stemness, cell proliferation, and invasion, while inducing apoptosis and delaying cell cycle progression in pancreatic cancer cells. We identified *MET*, *CTSA*, and *ANO1* as potential target genes of miR-4711-5p. Furthermore, the administration of miR-4711-5p formulated with super carbonate apatite (sCA) did not show any apparent treatment-related adverse effects even at a supra-therapeutic dose in the cynomolgus monkey study. This study provides strong preclinical evidence supporting miR-4711-5p as a novel and safe therapeutic strategy for pancreatic cancer and represents an important step toward clinical application.

## 1. Introduction

Pancreatic cancer is a highly malignant neoplasm and is responsible for 467,005 cancer deaths annually, ranking sixth in mortality worldwide in 2022 [1]. For resectable pancreatic cancer, the standard first-line approach is curative surgical resection followed by adjuvant therapy. Among adjuvant regimens, modified FOLFIRINOX provides the greatest survival benefit in fit patients. For unresectable cases, standard chemotherapy consists of nab-paclitaxel and gemcitabine or the FOLFIRINOX combination approach, along with Poly ADP-ribose polymerase (PARP) inhibitors, is conducted [2]. However, these treatments have only shown minimal efficacy, and pancreatic cancer continues to have a poor prognosis, with a very low 5-year survival rate. Therefore, the development of novel and effective therapeutic strategies for pancreatic cancer remains an urgent unmet need.

The concept of cancer stem cells (CSCs) has been the focus of attempts to elaborate cancer cells in recent years. Their characteristics of self-renewal, multipotency, tumorigenic potential, and resistance to therapy are considered to be the cause of recurrence and metastasis; therefore, targeting CSCs may lead to a radical cure for cancer [3,4]. In pancreatic cancer, various molecules are considered CSC markers, including CD133, CD44, CD24, and ALDH [5]. KLF5 is one of the zinc finger transcription factors belonging to the Kruppel-like factor (KLF) family of proteins. The KLF family regulates gene expression by binding zinc finger domains to GC-rich sites in promoter and enhancer regions [6]. KLF5 is required for stemness in Embryonic Stem (ES) cells and may be a factor in the generation of induced pluripotent stem (iPS) cells [7]. KLF5 is also thought to play an oncogenic role in breast, bladder, lung, stomach, and colon cancer by promoting cell proliferation [8]. It is shown that KLF5 is highly expressed in colorectal cancer cells and associated with cancer stemness [9,10]. In pancreatic cancer, KLF5 has been reported to be upregulated and is associated with poor prognosis and tumorigenesis. Furthermore, its overexpression correlates with G1/S cell cycle progression [11,12].

We previously showed that miR-4711-5p directly targets *KLF5* mRNA and suppresses cancer stemness, cell proliferation, migration, and invasion activity in colorectal cancer cells. Furthermore, miR-4711-5p targets *TFDP1* and *MDM2* mRNA, which may contribute to the induction of G1 arrest and apoptosis [13]. Although these findings highlighted the therapeutic potential of miR-4711-5p, its relevance in pancreatic cancer and its translational applicability have not been fully elucidated. To our knowledge, there are no previous reports demonstrating the direct therapeutic potential of miR-4711-5p in pancreatic cancer. In addition to previously identified targets, this study also aims to explore novel downstream targets of miR-4711-5p that may contribute to its antitumor effects.

Importantly, while our previous study demonstrated the antitumor efficacy and lack of significant toxicity of miR-4711-5p in mouse models, its safety profile in higher-order species remains unknown. Therefore, as a step toward clinical translation, we conducted a pilot preclinical safety assessment using non-human primates. This approach provides critical insights into the feasibility and safety of miR-4711-5p for future therapeutic applications.

The purpose of this study is to clarify the feasibility of miR-4711 as a potential therapeutic option against pancreatic cancer.

## 2. Materials and Methods

### 2.1. Cell Culture

All cells were obtained from the American Type Culture Collection (ATCC). Human pancreatic cancer cell lines SUIT-2 and BxPC-3 were cultured as previously described [14].

### 2.2. Transfection of miRNA

Transfection of miRNA was performed as previously described [14]. All miRNAs used in this study were obtained from GeneDesign, Inc. (Osaka, Japan):

miR-4711-5p, S: 5′-UGCAUCAGGCCAGAAGACAUGAG-3′

AS: 5′-CUCAUGUCUUCUGGCCUGAUGCA-3′

Negative control RNA (NC), S: 5′-AUCCGCGCGAUAGUACGUA-3′

AS: 5′-UACGUACUAUCGCGCGGAU-3′

Negative control RNA 2 (NC2) was purchased from Sigma Aldrich (St. Louis, MO, USA) (MISSION^®^ siRNA Universal Negative Control #1).

### 2.3. Western Blotting

Western blotting was performed as previously described [15]. The primary antibodies used in this study were anti-β-actin antibody (1:1000, Cell Signaling Technology, Danvers, MA, USA), anti-KLF5 antibody (1:200, R&D SYSTEMS, Minneapolis, MN, USA), and anti-TFDP1 antibody (1:100, Thermo Fisher Scientific, Inc., Waltham, MA, USA). Bands were detected by the ImageQuant LAS 4000mini (GE Healthcare, Chicago, IL, USA).

### 2.4. Quantitative RT-PCR

Cells were lysed in TRIzol reagent (Thermo Fisher Scientific, Inc.) and total RNA extracted by following the standard protocol. Complementary DNA was generated from RNA using the High-Capacity cDNA Reverse Transcription Kit (Thermo Fisher Scientific, Inc.). We performed qPCR using the LightCycler 480 System II (Roche Diagnostics, Rotkreuz, Switzerland). Relative expression was quantified using the ΔΔCt method [16]. Each value was normalized to GAPDH expression. The primer sequences are listed in Supplementary Table S1.

### 2.5. Water-Soluble Tetrazolium (WST) Assay

Cell proliferation was assessed by the WST-1 assay as previously described [14]. A total of 3.0–4.0 × 10^3^ cells were plated in 96-well plates and treated with miRNA. We added 10 μL of Cell Counting Kit solution (Dojindo Molecular Technologies, Inc., Kumamoto, Japan) to each well 24, 48, and 72 h after transfection.

### 2.6. Matrigel Invasion Assay

Matrigel invasion assay was performed as previously described [14]. SUIT-2, or BxPC-3 cells, were seeded into the upper chambers at a density of 7.5 × 10^4^ cells per chamber in the appropriate medium containing 0.1% bovine serum albumin. Medium containing 10% FBS was added to the lower wells. Cells were transfected with the miRNAs and incubated at 37 °C after transfection. After incubation for 48 or 72 h, invaded cells were fixed and stained with hematoxylin.

### 2.7. Bromodeoxyuridine (BrdU) Proliferation Assay

BrdU proliferation assay was performed as previously described [15]. A total of 3.0 × 10^4^ cells were plated in a 96-well plate and treated with miRNAs. Cells were cultured with BrdU labeling reagent for 3 h at 37 °C, incubated with anti-BrdU monoclonal antibody for 1 h at room temperature, and then incubated with HRP-conjugated secondary antibody for 1 h at room temperature. After the addition of the substrate reagent, the absorbance in each well was measured.

### 2.8. Annexin V Assay

Annexin V assay was performed as previously described [14]. A total of 2.0 × 10^5^ cells were plated and treated with miRNAs. The population of apoptotic cells was assessed by flow cytometry using Spectral Analyzer SA3800 (Sony Biotechnology, Inc., San Jose, CA, USA). Annexin V-positive and Propidium iodide (PI)-negative cells were defined as early apoptosis. Annexin V-positive and PI-positive cells were defined as late apoptosis.

### 2.9. Sphere Formation Assay

The sphere formation assay was performed as previously described [13]. SUIT-2 or BxPC-3 cells were seeded in 96-well ultralow-attachment plates (Corning Inc., Corning, NY, USA) at a density of 1000 or 5000 cells per well, respectively. Cells were treated with miRNA simultaneously. We counted the number of spheres ≥40 μm 4 days after seeding or ≥80 μm 7 days after seeding for BxPC-3 and SUIT-2 cells, respectively.

### 2.10. Production of Super Carbonate Apatite (sCA)

After adding 40 mL of CaCl_2_ solution containing 120 mg of miR-4711-5p to 10 L of inorganic solution (44 mM NaHCO_3_; 0.9 mM NaH_2_PO_4_; 1.8 mM CaCl_2_, pH 7.5) pre-warmed to 37 degrees, it was incubated for 7 min, and the reaction was stopped with 5 L stop solution (0.3% carmellose). The solution was centrifuged at 12,000 rpm for 5 min. The precipitate was suspended in injectable water to reach the concentration required for toxicity experiments.

### 2.11. Non-Human Primate Safety Evaluation

A single-dose intravenous toxicity study was conducted in male cynomolgus monkeys (*Macaca fascicularis*). Six quarantine-cleared animals were initially received, and a 14-day acclimatization period was conducted prior to group allocation. During acclimatization, general clinical observations were performed once daily. Animals were habituated to the dosing procedure by restraint on Days −6, −4, and −2. Four animals that met predefined health criteria were allocated to the study groups (body weight at group allocation: 3.91–4.10 kg). No animals were excluded after group allocation.

Animals were housed two per cage in stainless steel cages under controlled environmental conditions (temperature 24.9–28.1 °C; relative humidity 43–76%; 12-h light/dark cycle). Animals were provided with a standard primate diet (Purina Mills, Gray Summit, MO, USA) and had free access to water. Environmental enrichment, including toys and fruit supplements, was provided. The experimental unit was a single animal.

Animals were allocated to two groups (*n* = 2 per group): a control group receiving nucleic acid–free super carbonate apatite (sCA) and a treatment group receiving miR-4711-5p formulated with sCA at 7.5 mg/kg (as nucleic acid content). This concentration is calculated based on human equivalent dose (HED) [17] and is estimated to be 10 times higher than the standard therapeutic dose for humans. Randomization and blinding were not performed. The dosing solution was administered intravenously via the cephalic vein at an infusion rate of 1 mL/min (injection volume 1.67 mL/kg).

The sample size was determined based on an exploratory pilot toxicology design. No formal a priori sample size calculation was performed.

General clinical observations were conducted during the acclimatization and post-dose periods. Body weight was measured at the start and end of acclimatization, prior to dosing, on Days 3 and 6 post-dosing, and at necropsy. Food consumption was recorded daily from Day −7. Ophthalmological examinations were performed on Day −4 and Day 3. Electrocardiography and blood pressure measurements were conducted on Day −6 and Day 3. Blood samples for hematology, coagulation, and serum biochemistry were collected on Day −6 and Day 3.

All animals were necropsied on the day following completion of the observation period. Animals were anesthetized by intramuscular administration of ketamine (Ketalar, 50 mg/mL, 0.3 mL/kg; Daiichi Sankyo Propharma Co., Ltd., Tokyo, Japan) in combination with medetomidine hydrochloride (Domitor, 1 mg/mL, 0.08 mL/kg; Orion Corporation, Espoo, Finland), followed by exsanguination. A full macroscopic examination was performed. Absolute organ weights were measured and relative organ weights were calculated. Organs and tissues were fixed in 10% neutral-buffered formalin, processed, and stained with hematoxylin and eosin for histopathological evaluation.

### 2.12. RNA Sequencing

We performed RNA sequencing as described previously [18]. The library was prepared using a TruSeq Stranded mRNA Sample Prep Kit (Illumina, San Diego, CA, USA). Sequencing was performed using the Illumina HiSeq 2500 platform in 75-base single-end mode. Illumina Casava 1.8.2 software was used for base calling, and the sequenced reads were mapped to human reference genome sequences (hg19) using TopHat version 2.0.13 combined with Bowtie2 version 2.2.3 and SAMtools version 0.1.19. We calculated the fragments per kilobase of exon per million mapped fragments (FPKM) using Cuffnorm version 2.2.1.

### 2.13. Data Analysis

Data analysis was performed using Kaplan–Meier Plotter (“https://kmplot.com/analysis/index.php?p=service&cancer=pancancer_rnaseq (accessed on 25 February 2026)”), TargetScan (“http://www.targetscan.org/ (accessed on 25 February 2026)”), miRBase (“http://www.mirbase.org/ (accessed on 25 February 2026)”), iDEP (“http://bioinformatics.sdstate.edu/idep/ (accessed on 25 February 2026)”), and Cancer Cell Line Encyclopedia (CCLE; “https://sites.broadinstitute.org/ccle/ (accessed on 25 February 2026)”).

### 2.14. Statistical Analysis

All data are given as means ± standard deviations. Student’s *t* test and Mann–Whitney U test were used to calculate the statistical significance. *p* < 0.05 was considered significant.

## 3. Results

### 3.1. miR-4711-5p Suppresses KLF5 Expression and Cancer Stemness in Pancreatic Cancer Cells

We previously reported that miR-4711-5p exhibits antitumor effects in colorectal cancer cells by regulating cancer stemness and the cell cycle, and identified KLF5 as a direct target of miR-4711-5p [13]. Therefore, we investigated whether miR-4711-5p treatment affects KLF5 expression and cancer stemness in pancreatic cancer cell lines.

**Figure 1:**
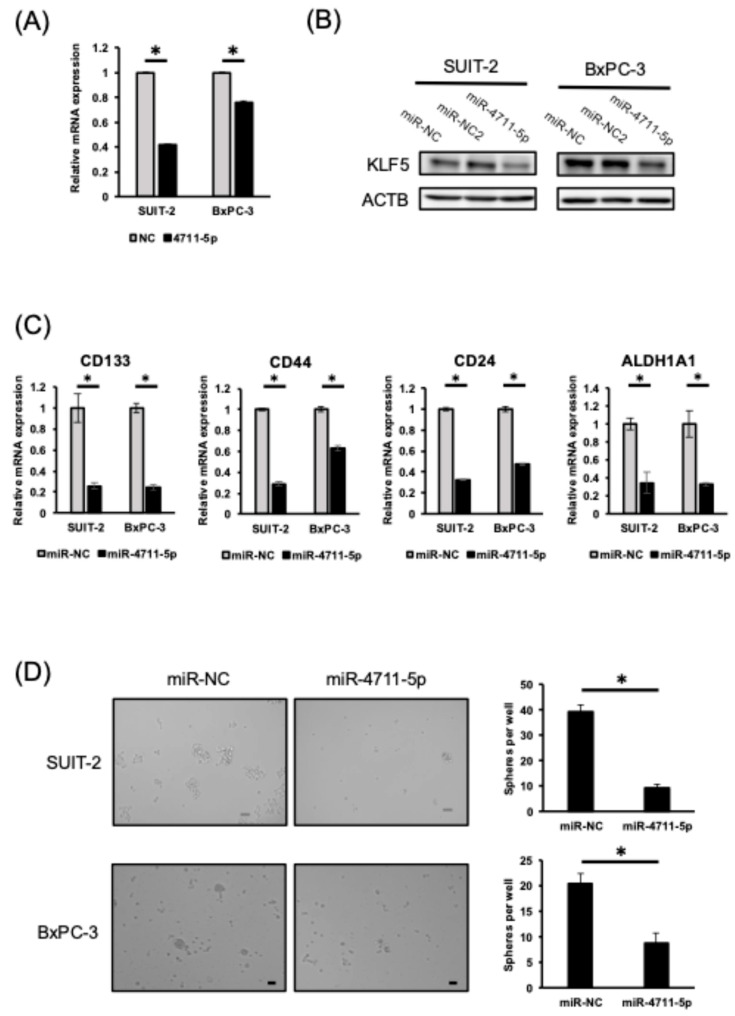
Effects of miR-4711-5p on KLF5 expression and stem cell properties in pancreatic cancer cell lines. (**A**,**B**) MiR-4711-5p treatment suppressed mRNA (**A**) and protein (**B**) expression of *KLF5* in SUIT-2 and BxPC-3 cells. (**C**) MiR-4711-5p treatment suppressed the expression of stem cell markers in pancreatic cells. (**D**) MiR-4711-5p treatment suppressed the sphere formation activity in pancreatic cells. Scale bar: 80 µm (SUIT-2) or 40 µm (BxPC-3). All experiments were performed in triplicate (*n* = 3, technical replicate for qPCR and sphere formation assay). All data are presented as the mean ± SD. * *p* < 0.01 (Student’s *t* test). Original western blots are presented in Figure S5.

According to publicly available Kaplan–Meier Plotter analysis of 177 cases, high KLF5 expression was significantly associated with poorer overall survival in patients with pancreatic cancer, supporting its oncogenic role (Supplementary Figure S1). Based on CCLE database analysis, we selected the SUIT-2 and BxPC-3 cell lines for the experiments because they showed higher *KLF5* mRNA expression than Panc-1 cells (Supplementary Figure S2). MiR-4711-5p treatment suppressed *KLF5* mRNA and protein expression in pancreatic cancer cells, similar to the results observed in colorectal cancer cells (Figure 1A,B). Furthermore, miR-4711-5p treatment decreased the expression of cancer stem cell (CSC) markers such as *CD133*, *CD44*, *CD24*, and *ALDH1A1*, and suppressed sphere-forming ability in pancreatic cancer cells (Figure 1C,D).

### 3.2. miR-4711-5p Suppresses Cell Proliferation and Cell Cycle Progression by Decreasing the Expression of TFDP1 and Pre-Replication Complex Genes in Pancreatic Cancer Cells

**Figure 2:**
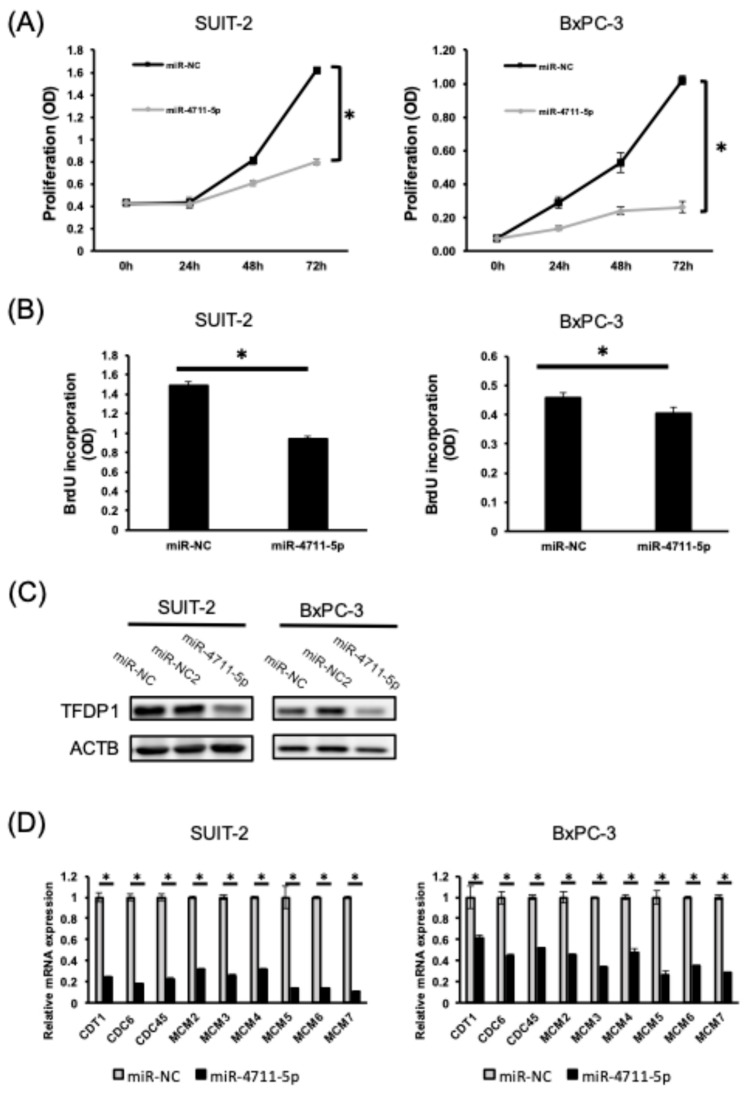
Effects of miR-4711-5p on cell proliferation, cell cycle, and the expression of cell cycle-related molecules in pancreatic cancer cell lines. (**A**) MiR-4711-5p treatment suppressed cell proliferation in SUIT-2 and BxPC-3 cells. (**B**) MiR-4711-5p treatment suppressed BrdU incorporation in SUIT-2 and BxPC-3 cells. (**C**) MiR-4711-5p suppressed the expression of TFDP1 protein in pancreatic cancer cell lines. (**D**) MiR-4711-5p suppressed the expression of pre-replication complex genes. All experiments were performed in triplicate (*n* = 3, technical replicate for qPCR, WST-1 assay and BrdU assay). All data are presented as the mean ± SD. * *p* < 0.01 (Student’s *t* test). Original western blots are presented in Figure S5.

As we previously showed that TFDP1 is a direct target of miR-4711-5p, and miR-4711-5p treatment decreased the expression of pre-replication complex genes in colorectal cancer cells [13], we examined the effect of miR-4711-5p treatment on cell proliferation and cell cycle progression in pancreatic cancer cells. MiR-4711-5p treatment suppressed cell proliferation and BrdU incorporation (Figure 2A,B) and decreased TFDP1 and pre-replication complex genes in the pancreatic cancer cells (Figure 2C,D). These results suggest that miR-4711-5p treatment prevented the cell cycle transition from G1 to S phase by decreasing the expression of TFDP1 and pre-replication complex genes.

### 3.3. miR-4711-5p Induces Apoptosis and Suppresses Invasion of Pancreatic Cancer Cells

**Figure 3:**
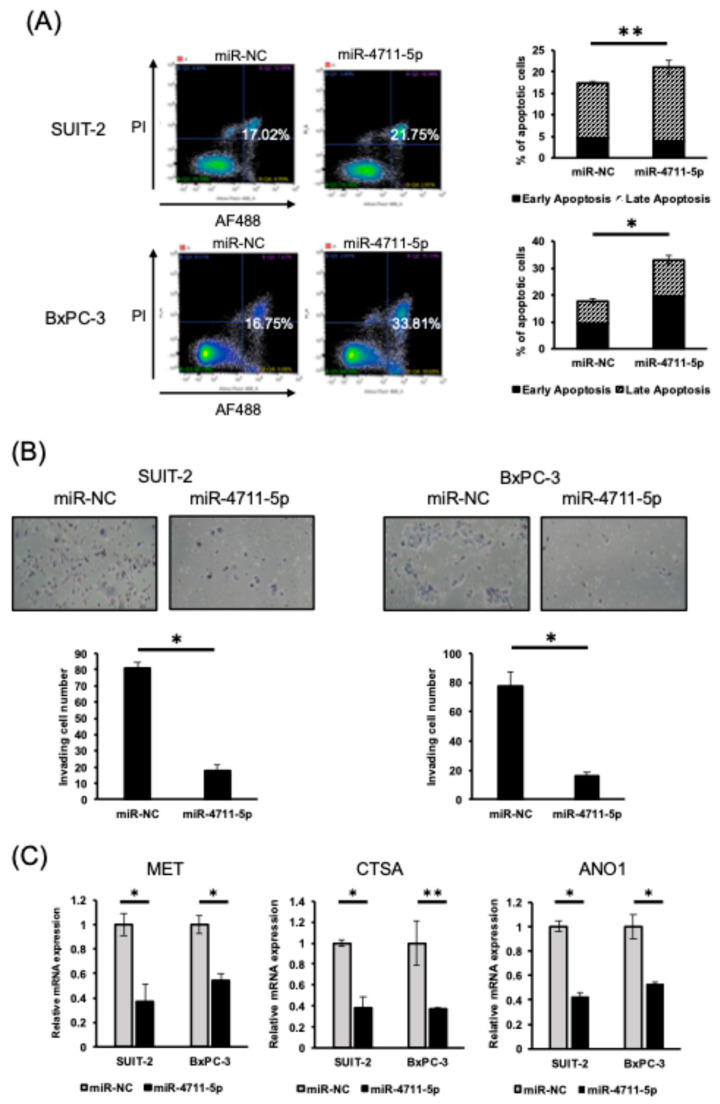
Effects of miR-4711-5p on apoptosis, invasive activity, and potential target gene expression in pancreatic cancer cell lines. (**A**) MiR-4711-5p treatment induced apoptosis in SUIT-2 and BxPC-3 cells. Representative images are shown (left). Percentages indicate the sum of early apoptotic cells (annexin V (AF488)-positive and propidium iodide (PI)-negative) and late apoptotic cells (annexin V-positive and PI-positive). (**B**) MiR-4711-5p treatment suppressed the invasive activity of SUIT-2 and BxPC-3 cells. Cells were seeded at a density of 7.5 × 10^4^ cells/chamber, and the invaded cells were counted 48 h after transfection; 400× magnification. (**C**) MiR-4711-5p suppressed the expression of potential target genes *MET*, *CTSA*, and *ANO1*. All experiments were performed in triplicate (*n* = 3, technical replicate for qPCR, annexin assay and matrigel invasion assay). All data are presented as the mean ± SD. * *p* < 0.01, ** *p* < 0.05 (Student’s *t* test).

Next, we investigated the effects of miR-4711-5p treatment on apoptosis and invasiveness of pancreatic cancer cells. The apoptosis assay demonstrated that treatment with miR-4711-5p induced apoptosis, while the Matrigel invasion assay showed that it markedly suppressed invasive activity in both SUIT-2 and BxPC-3 cell lines. (Figure 3A,B).

### 3.4. MET, CTSA, and ANO1 Genes Are Potential Targets of miR-4711-5p

To identify novel potential targets of miR-4711-5p, we performed RNA sequencing and compared gene expression profiles between control and miR-4711-5p–treated BxPC-3 cells 36 h after transfection. We analyzed the RNA sequencing data (Supplementary Figure S3) and performed in silico analysis with TargetScan to select the potential target genes that met the following criteria: expression decreased >1.75-fold with miR-4711-5p treatment, average FPKM of parent sample > 10, and the mRNA possessed the binding site of the miR-4711-5p seed sequence. We confirmed that *KLF5* and *TFDP1* satisfied these criteria, and we identified *MET*, *CTSA*, and *ANO1* as the potential target genes of miR-4711-5p (Supplementary Figure S3). We performed qPCR and found that miR-4711-5p treatment suppressed the expression of *MET*, *CTSA*, and *ANO1* in the pancreatic cancer cells (Figure 3C).

### 3.5. Safety Evaluation of miR-4711-5p in Cynomolgus Monkeys

**Figure 4:**
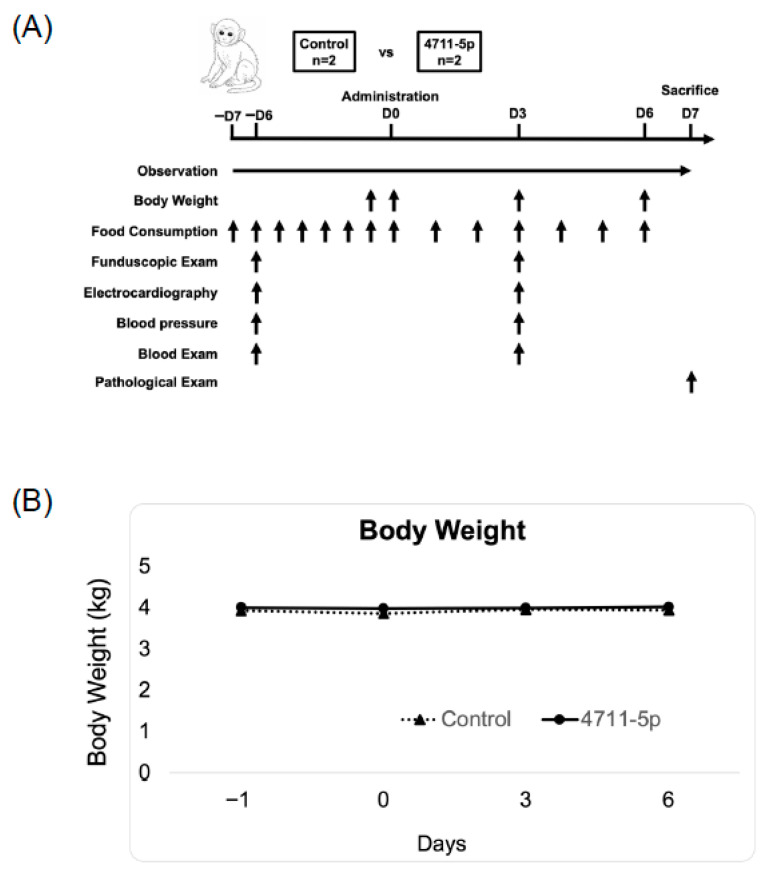
Non-human safety evaluation. (**A**) Schematic overview of the safety study using cynomolgus monkeys. A total of four cynomolgus monkeys were assigned (*n* = 2 each) to the control group or the miR-4711-5p–treated group. General clinical conditions were monitored daily from Day −7 to Day 6 (arrows). Body weight was measured on Days −1, 0, 3, and 6, and daily food consumption was recorded throughout the observation period. Funduscopic examination, electrocardiography, blood pressure measurement, and blood examinations were performed on Days −6 and 3. On Day 7, all animals were sacrificed, and histopathological examinations were conducted. (**B**) Changes in body weight. Body weight was measured on Days −1, 0,3, and 6. Data are shown as mean values for the control group (*n* = 2) and 4711-5p group (*n* = 2).

By using the mouse model, we previously showed the systemic safety of miR-4711-5p formulated with sCA, which is a pH-sensitive delivery system for miRNA and siRNA with no significant immune activation [13,19]. As part of further research toward clinical application, a single-dose intravenous toxicity study was conducted in cynomolgus monkeys to assess the systemic safety of miR-4711-5p formulated with sCA. An overview of the study design and the time course of evaluations is shown in Figure 4A. No treatment-related changes in body weight were observed in the miR-4711-5p–treated group compared with the control group throughout the observation period (Figure 4B). Body weight remained stable in all animals from Day −1 to Day 6 following administration. Hematological parameters, including red and white blood cell counts, hemoglobin levels, platelet counts, and coagulation-related indices, showed no clinically relevant or treatment-related changes in the miR-4711-5p–treated animals compared with controls (Table 1). Similarly, blood biochemical analyses revealed no abnormalities attributable to the test article, and all measured parameters remained within physiological ranges (Table 1). Relative organ weights were comparable between the control and miR-4711-5p–treated groups, with no findings suggestive of treatment-related organ toxicity (Table 2). No mortality or abnormal clinical signs were observed in either group during the study period. Food consumption showed no changes considered to be related to miR-4711-5p administration. Ophthalmologic examinations revealed no abnormalities in any animal. Electrocardiographic parameters and blood pressure measurements showed no treatment-related changes. At necropsy, no gross pathological abnormalities were observed. Histopathological examination of major organs revealed no lesions or findings considered to be related to miR-4711-5p administration. Collectively, these results indicate that intravenous administration of miR-4711-5p formulated with sCA did not induce any detectable adverse effects in cynomolgus monkeys, even at a dose equivalent to 10-fold the effective dose used in prior mouse efficacy studies.

## 4. Discussion

MiRNAs are involved in post-transcriptional regulation of gene expression and have been studied extensively for cancer therapy because they can simultaneously regulate multiple target genes and exert antitumor effects through multimolecular regulatory mechanisms [20,21]. Consistent with our previous findings in colorectal cancer cells [13], miR-4711-5p suppressed *KLF5* expression at both the mRNA and protein levels in pancreatic cancer cells. In addition, miR-4711-5p treatment reduced the expression of cancer stem cell (CSC)–associated markers and suppressed sphere-forming ability. Although the role of *KLF5* in pancreatic cancer stemness has not been fully elucidated, our findings suggest that miR-4711-5p suppresses pancreatic cancer stemness through *KLF5* inhibition, similar to its effects in colorectal cancer.

We further demonstrated that miR-4711-5p suppressed cell proliferation and delayed cell cycle progression in pancreatic cancer cells through the downregulation of *TFDP1* and multiple genes involved in the pre-replication initiation complex. These results indicate that miR-4711-5p interferes with G1-to-S phase transition via *TFDP1* suppression, consistent with our previous observations in colorectal cancer cells [13]. We also previously showed that miR-4711-5p suppressed the expression of G1/S checkpoint components including CyclinD1, CDK2, 4, and 6 in colorectal cancer cells [13]. RNA-seq data showed that miR-4711-5p modestly suppressed the expression of CyclinD1 and CDK4 in BxPC-3 (Supplementary Figure S4). Although further detailed experiments are required, this may also be associated with cell cycle delay by miR-4711-5p treatment. Taken together, these findings suggest that miR-4711-5p exerts antitumor effects in pancreatic cancer by coordinately targeting cancer stemness and cell cycle–associated pathways.

Although this study was limited to two cell lines, we intend to advance our research toward clinical application by conducting future studies using other cell lines and clinical specimens (e.g., Patient-derived xenograft models).

One of the critical challenges in nucleic acid medicine is the development of a miRNA delivery system that ensures sufficient stability of therapeutic miRNAs while enabling efficient systemic distribution without inducing toxicity or off-target effects [22,23,24]. To address this issue, we developed a drug delivery system based on sCA, which enables more efficient delivery compared to other non-viral vectors, such as liposomes and atelocollagen [19]. We showed that sCA successfully delivered various nucleic acids or low molecular weight reagents in tumor or IBD (inflammatory bowel disease) model mice without apparent abnormalities in mice [15,18,25,26]. Furthermore, a previous study demonstrated that systemic administration of miR-4711-5p formulated with sCA suppressed the growth of established colorectal cancer xenografts in nude mice without significant adverse effects. These findings support the feasibility of miR-4711-5p–based therapy using an optimized delivery system. This DDS has been repeatedly described as a promising non-viral vector in review articles [27,28,29,30].

We newly identified some potential targets of miR-4711-5p and focused on *MET, CTSA*, and *ANO1. MET* encodes an HGF receptor and is a well-known oncogene. Overexpression of *MET* has been reported to be associated with poor prognosis in lung, breast, colon, gastric, and pancreatic cancers [31]. Activation of MET signaling triggers multiple downstream pathways, including the RAS/RAF/MEK/ERK and PI3K/AKT pathways, which promote cell proliferation and survival while suppressing apoptosis [32,33]. Through these mechanisms, aberrant MET signaling contributes to tumor progression and malignant phenotypes. Therefore, suppression of MET by miR-4711-5p may attenuate these oncogenic signaling pathways, leading to reduced proliferation and potentially promoting apoptotic responses in pancreatic cancer cells. *ANO1* is a Ca^2+^-activated Cl^−^ channel (CaCC) expressed in peripheral somatosensory neurons and activated by painful stimuli. In pancreatic cancer, high expression of the *ANO1* gene has been reported to be associated with poor prognosis [34,35]. ANO1 has also been implicated in cancer progression and has been reported to enhance tumor growth and invasiveness through activation of ERK and PI3K signaling pathways [36,37]. These observations suggest that simultaneous repression of MET and ANO1 by miR-4711-5p may cooperatively attenuate common proliferative and survival pathways. *CTSA* is a lysosomal protease that is highly expressed in cancer cells that have acquired metastatic potential and degrades basement membrane and extracellular matrix components. *CTSA* has been reported to promote epithelial–mesenchymal transition (EMT) in prostate cancer cell lines [38]. Cathepsins have been implicated in tumor invasion and metastasis through degradation of the extracellular matrix and remodeling of the tumor microenvironment [39]. Thus, downregulation of CTSA by miR-4711-5p may provide an additional mechanism contributing to the anti-tumor effects of miR-4711-5p, potentially through suppression of invasive phenotypes. Taken together, the down-regulation of these molecules could also be associated with antitumor effects of miR-4711-5p.

Importantly, we extended the preclinical evaluation of miR-4711-5p to a non-human primate model and demonstrated that a single intravenous administration of miR-4711-5p formulated with sCA did not induce treatment-related abnormalities in clinical observations, laboratory parameters, organ weights, or histopathological findings, even at a dose equivalent to 10-fold the effective dose used in mouse efficacy studies. A limitation of this study is that, due to the small sample size (*n* = 2), its statistical robustness is limited. This was attributed mainly to the high cost of monkeys, and instead, we administered a 10-fold higher dose of miR-4711-5p and tried to find out a subtle and ominous sign, if any. However, given the limited availability of non-human primate safety data for systemically administered miRNA therapeutics, these findings provide important preclinical safety information and represent a meaningful step toward the future clinical application of miRNA-based cancer therapies, although further studies with larger sample sizes will be required. Furthermore, the present study involved only in vitro experiments. Therefore, to validate these findings, future research will include in vivo experiments using pancreatic cancer mouse models to evaluate both the antitumor efficacy and in vivo biodistribution of miR-4711-5p formulated with sCA.

## 5. Conclusions

miR-4711-5p exhibits potent antitumor activity in pancreatic cancer cells by suppressing cancer stemness, inducing apoptosis, inhibiting cell cycle progression, and reducing invasive capacity through the regulation of multiple oncogenic pathways. Importantly, miR-4711-5p formulated with sCA showed a favorable safety profile in a non-human primate model, even at a supra-therapeutic dose. These findings support miR-4711-5p as a promising miRNA-based therapeutic candidate for pancreatic cancer.

## Figures and Tables

**Table 1:** Blood examination.

	Pre-Treatment (Day −6)				Post-Treatment (Day 3)					
	Control_1	Control_2	4711_5p_1	4711_5p_2	Control_1	Control_2	4711_5p_1	4711_5p_2	Control Student’s *t*-test *p* Value	4711_5p Student’s *t*-test *p* Value
Hematology										
RBC (10^4^/μL)	557	545	558	604	505	499	546	552	0.039	0.356
HGB (g/dL)	13.1	14.2	14.4	14.1	11.9	12.8	14	12.8	0.049	0.310
HCT (%)	42.5	44.6	44	44.8	39.5	41.6	44	44.8	NE	NE
MCV (fL)	76.3	81.8	78.9	74.2	78.2	83.4	80.6	75	0.054	0.220
MCH (pg)	23.5	26.1	25.8	23.3	23.6	25.7	25.6	23.2	0.656	0.205
MCHC (g/dL)	30.8	31.8	32.7	31.5	30.1	30.8	31.8	30.9	0.111	0.126
PLT (10^3^/μL)	392	426	452	362	324	364	320	350	0.029	0.442
Reticulocyte (%)	0.61	0.6	0.65	0.93	0.95	0.88	0.77	1.24	0.061	0.265
WBC (10^3^/μL)	13.51	11.34	13.17	12.14	10.83	12.16	12.34	15.27	0.689	0.665
NEUT (10^3^/μL)	3.88	3.7	3.31	3.13	3.48	3.88	3.61	5.22	0.769	0.409
LYMP (10^3^/μL)	8.78	6.86	8.98	8.28	6.53	6.84	7.44	8.39	0.494	0.545
MONO (10^3^/μL)	0.62	0.55	0.49	0.5	0.64	1.08	1.03	1.42	0.476	0.162
EOS (10^3^/μL)	0.21	0.2	0.38	0.2	0.12	0.28	0.22	0.17	0.963	0.382
BASO (10^3^/μL)	0.02	0.03	0.01	0.03	0.06	0.08	0.04	0.07	0.070	0.090
Coagulation										
PT (s)	10.4	10.2	10.5	10.7	10.3	9.7	10.3	10.5	0.374	NE
APTT (s)	19.8	17.9	22.6	18.6	21.9	18	23.2	19.4	0.470	0.090
Chemistry										
AST (IU/L)	29	31	27	24	34	47	35	28	0.307	0.205
ALT (IU/L)	23	39	25	33	39	77	41	47	0.246	0.042
ALP (IU/L)	981	1965	1305	1574	1140	1782	1220	1415	0.955	0.187
CK (IU/L)	128	150	191	122	245	198	230	173	0.252	0.084
T_BIL (mg/dL)	0.06	0.11	0.04	0.07	0.08	0.08	0.06	0.1	0.874	0.126
TP (g/dL)	6.7	7.7	6.8	7.5	6.9	7.6	6.9	7.5	0.795	0.500
ALB (g/dL)	3.8	3.9	3.8	4.2	3.7	3.7	3.8	4.1	0.205	0.500
Globulin (g/dL)	2.9	3.8	3	3.3	3.2	3.9	3.1	3.4	0.295	NE
A/G	1.31	1.03	1.27	1.27	1.16	0.95	1.23	1.21	0.188	0.126
TG (mg/dL)	34	44	26	60	54	100	51	100	0.282	0.144
T_CHO (mg/dL)	109	153	109	87	101	150	114	99	0.272	0.249
GLU (mg/dL)	65	87	81	83	76	74	74	90	0.947	1.000
BUN (mg/dL)	22.6	16.1	18.8	22.4	21.5	19.9	19.4	23.8	0.679	0.242
CRNN (mg/dL)	0.73	0.68	0.57	0.68	0.76	0.7	0.64	0.67	0.126	0.590
P (mg/dL)	5.93	6.35	6.58	4.77	4.69	5.02	4.96	3.96	0.022	0.205
Ca (mg/dL)	9.9	10.1	10	9.9	9.5	9.9	9.7	10.1	0.205	0.874
Na (mmol/L)	145	148	145	143	149	148	148	145	0.500	0.126
K (mmol/L)	4.6	4.4	4.8	4	4.4	5.2	4.9	4.7	0.656	0.410
Cl (mmol/L)	110	107	105	109	110	108	107	111	0.500	NE

A/G: Albmin/Globlin ratio; NE: not estimable; the *p* value was not calculated because the paired differences were identical (zero variance); Student’s *t* test was performed (*n* = 2).

**Table 2:** Individual relative organ weight.

Animal No.	Body Weight (kg)	Pancreas (g/kg)	Spleen (g/kg)	Brain (g/kg)	Heart (g/kg)	Lung (g/kg)	Liver (g/kg)	Kidney_R (g/kg)	Kidney_L (g/kg)	Kidney_R + L (g/kg)
Control_1	3.87	1.19	1.5	18.22	2.84	4.63	16.15	1.63	1.6	3.23
Control_2	3.91	1.25	1.28	16.5	3.58	4.65	17.8	1.94	1.97	3.91
4711-5p_1	3.74	1.93	1.12	18.29	3.32	5.27	18.8	2.01	1.98	3.98
4711-5p_2	4.04	1.78	1.01	16.68	3.49	4.98	19.33	1.71	1.91	3.61
MU *p* value	1.00	0.33	0.33	0.67	1.00	0.33	0.33	0.67	0.67	0.67

Mann–Whitney U test (MU) was performed (*n* = 2).

## Data Availability

The raw data were deposited in the NCBI Gene Expression Omnibus database under GEO accession number GSE305461. Data from the non-human primate safety study are available from the corresponding author upon reasonable request and subject to institutional and ethical regulations.

## Supplementary Materials

The following supporting information can be downloaded at: https://www.mdpi.com/article/10.3390/cancers18071104/s1, Figure S1: Patients with pancreatic cancer (*n* = 177) were divided into high and low KLF5 mRNA expression groups, and overall survival was compared using Kaplan–Meier analysis; Figure S2: *KLF5* mRNA expression in Panc-1, SUIT-2, and BxPC-3 cells; Figure S3: RNA sequencing results. miR-4711-5p treatment (36 h) suppressed the expression of MET, CTSA, and ANO1 in BxPC-3 cells; Figure S4: RNA sequencing results. miR-4711-5p treatment (36 h) modestly suppressed the expression of CCND1 and CDK4 in BxPC-3 cells; Figure S5: The original blot of Figure 1B (SUIT-2) and Figure 2C (BxPC-3). The band intensity of KLF5 and TFDP1 was normalized by ACTB. The relative ratio of each band is indicated; Table S1: Primer sequence for qRT-PCR.
