# Supplementary material for: Therapeutic Potential of miR-4711-5p in Pancreatic Cancer: Antitumor Activity and Mechanistic Insights
Source: Cancers (Basel). 2026 Mar 29;18(7):1104. doi: 10.3390/cancers18071104 (PMC13071966; doi:10.3390/cancers18071104)
Supplement: Supplementary file 1 [file cancers-18-01104-s001.zip › supplementary figures.pdf]

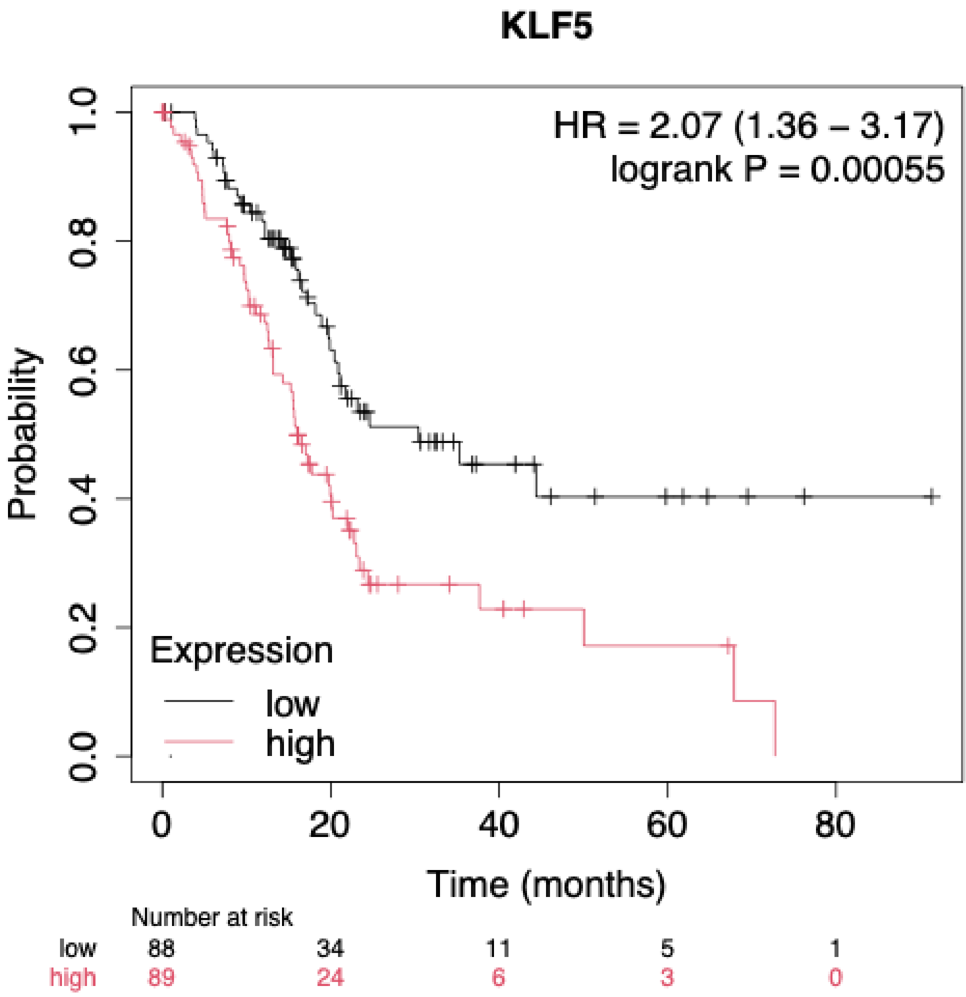

Supplementary Fig. S1
Patients with pancreatic cancer (n = 177) were divided into high and low KLF5 mRNA expression groups, and overall survival was compared using Kaplan–Meier analysis.

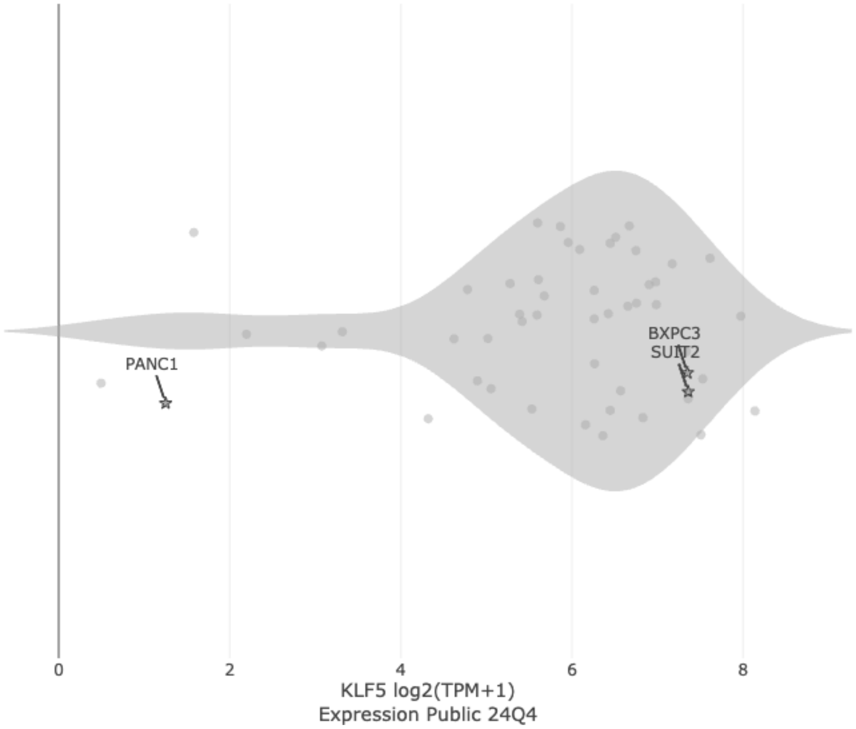

Supplementary Fig. S2
KLF5 mRNA expression in Panc-1, SUIT-2, and BxPC-3 cells.

Supplementary Figure S2

	miR-4711-5p vs Parent	miR−4711−5p		NC−1		Parent	
gene name	Fold Change	miR−4711−5p ①	miR−4711−5p ②	NC−1 ①	NC−1 ②	Parent ①	Parent ②
PSMB6	-4.603						
CHST14	-4.030						
CDH1	-3.903						
⋮	⋮	⋮		⋮		⋮	
**CTSA**	-2.437						
**TFDP1**	-2.306						
⋮	⋮	⋮		⋮		⋮	
**ANO1**	-2.210						
⋮	⋮	⋮		⋮		⋮	
**MET**	-2.107						
⋮	⋮	⋮		⋮		⋮	
**KLF5**	-1.896						
SLC7A1	-1.896						

Supplementary Fig. S3
RNA sequencing results. miR-4711-5p treatment (36 h) suppressed the expression of MET, CTSA, and ANO1 in BxPC-3 cells.

	miR-4711-5p vs Parent	miR-4711-5p		NC-1		Parent	
gene name	Fold Change	miR-4711-5p ①	miR-4711-5p ②	NC-1 ①	NC-1 ②	Parent ①	Parent ②
CCND1	-1.346						
CDK2	-1.023						
CDK4	-1.308						
CDK6	1.511						

Supplementary Fig. S4
RNA sequencing results. miR-4711-5p treatment (36 h) modestly suppressed the expression of CCND1 and CDK4 in BxPC-3 cells.

Supplementary Figure S4

(A)

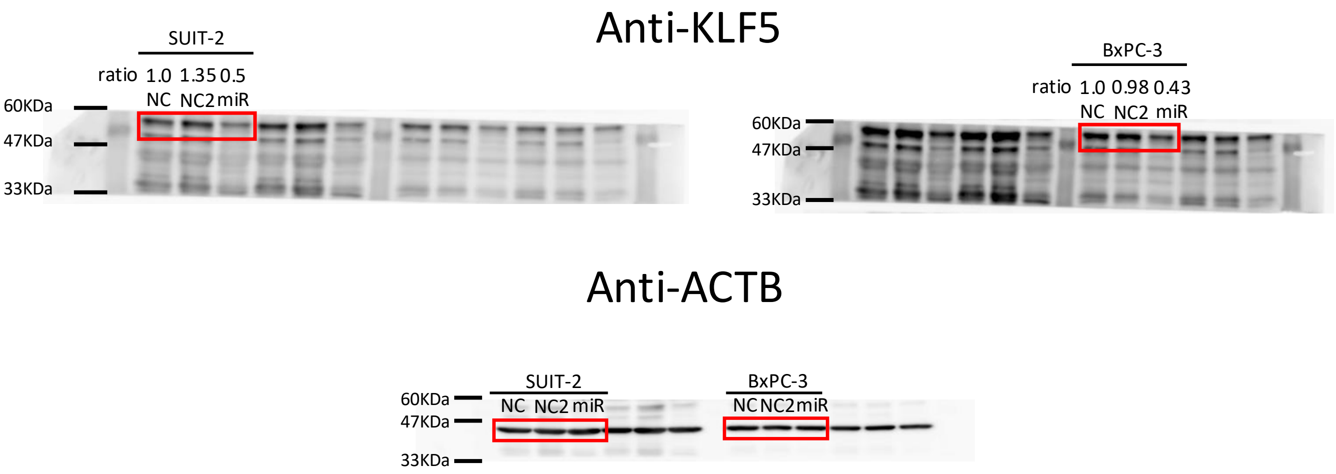

(B)

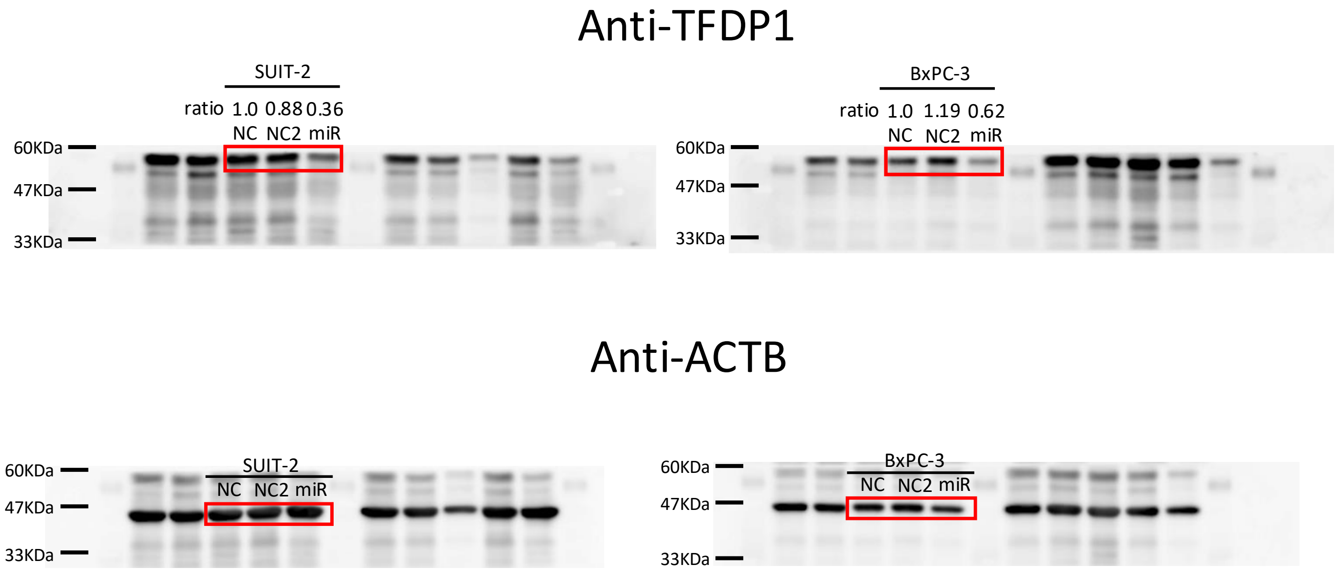

Supplementary Fig. S5
The original blot of Figure.1B (SUIT-2) and Figure. 2C (BxPC-3). The band
intensity of KLF5 and TFDP1 was normalized by ACTB. The relative ratio of each band is indicated.
